# Lycium barbarum polysaccharide encapsulated Poly lactic-co-glycolic acid Nanofibers: cost effective herbal medicine for potential application in peripheral nerve tissue engineering

**DOI:** 10.1038/s41598-018-26837-z

**Published:** 2018-06-06

**Authors:** Jing Wang, Lingling Tian, Liumin He, Nuan Chen, Seeram Ramakrishna, Kwok-Fai So, Xiumei Mo

**Affiliations:** 1 State Key Laboratory of Modification of Chemical Fibers and Polymer Materials, College of Chemistry, Chemical Engineering and Biotechnology, Donghua University, Shanghai, 201620 China; 2 Center for Nanofibers and Nanotechnology, E3-05-14, Department of Mechanical Engineering, Faculty of Engineering, National University of Singapore, 2 Engineering Drive 3, Singapore, 117576 Singapore; 3 Key Laboratory of Biomaterials of Guangdong Higher Education Institutes, Department of Biomedical Engineering, College of Life Science and Technology, Jinan University, Guangzhou, 510632 China; 4 Guangdong-Hongkong-Macau Institute of CNS Regeneration (GHMICR), Jinan University, Guangzhou, 510632 China; 5 Ministry of Education CNS Regeneration Collaborative Joint Laboratory, Jinan University, Guangzhou, 510632 China

## Abstract

Nerve regeneration is a serious clinical challenge following peripheral nerve injury. Lycium barbarum polysaccharide (LBP) is the major component of wolfberry extract, which has been shown to be neuroprotective and promising in nerve recovery in many studies. Electrospun nanofibers, especially core-shell structured nanofibers being capable of serving as both drug delivery system and tissue engineering scaffolds, are well known to be suitable scaffolds for regeneration of peripheral nerve applications. In this study, LBP was incorporated into core-shell structured nanofibrous scaffolds via coaxial electrospinning. Alamar blue assays were performed to investigate the proliferation of both PC12 and Schwann cells cultured on the scaffolds. The neuronal differentiation of PC12 cells was evaluated by NF200 expression with immunostaining and morphology changes observed by SEM. The results indicated that the released LBP dramatically enhanced both proliferation and neuronal differentiation of PC12 cells induced by NGF. Additionally, the promotion of Schwann cells myelination and neurite outgrowth of DRG neurons were also observed on LBP loaded scaffolds by LSCM with immunostaining. In summary, LBP, as a drug with neuroprotection, encapsulated into electrospun nanofibers could be a potential candidate as tissue engineered scaffold for peripheral nerve regeneration.

## Introduction

Peripheral nerve injury (PNI) caused by accidents, physical conflict and surgical intervention is a common global clinical problem which can significantly affect the patients’ quality of life. Over 200 thousand procedures were performed in the US alone annually to repair PNI, and it cost 150 billion dollars annually in the US, which cause an enormous socioeconomic burden^1^. Although the peripheral nervous system (PNS) has a capacity for axonal regeneration after injury, axonal reconnection and functional recovery by spontaneous peripheral nerve repair are nearly always incomplete.

In recent hundred years, various types of medical therapy have been used to repair nerve lesions. Normally, the surgical reconnection is possible for bridging small PNI gaps where the disconnected nerve stumps can be sutured end-to-end^2^. However, when the defect is larger (>10 mm in rats, or >30 mm in humans)^3^, retracts after injury and tensionless repair is impossible^4^. In this case, graft between nerve stumps is required to bridge the gap and support axonal regrowth. The implantation of autologous nerve graft is regard as the gold standard therapy for peripheral nerve gap repair^5^. However, several drawbacks of this therapy such as the need for multiple surgeries, and donor site morbidity limit its widespread clinical use, especially for longer nerve gaps. With the progress in tissue engineering, recent advances in neural tissue engineering have shown great promise for neural regeneration. Various biological and artificial nerve grafts have been used as supplement and even substitution for autologous nerve grafts. A typical tissue engineered nerve graft is composed a biomaterial-based scaffold and a multitude of cellular and/or molecular components^4^.

Generally, the scaffolds are capable of guiding the regeneration of axons and function as a bridge to restore the gap. Additionally, the biomaterial-based scaffold should mimic the native extracellular matrix (ECM) structure, which can provide enough space and adhesion sites for the growth of cells and extension of axons. What’s more, the scaffold should have the capacity to deliver drugs and/or signaling factors which can direct the growth and extension of regenerating axons.

Electrospun nanofibers have been used extensively as potential scaffold in neural tissue engineering^2^. It provides a conducive environment for cellular functions including adhesion, migration, proliferation and differentiation due to its close imitation of native neural ECM. On the other hand, core-shell structured nanofibers fabricated via coaxial electrospinning has frequently been served as a carrier for drug release application^6,7^. Proteins, drugs, and nucleic acids all have been delivered by core-shell nanofibers. The drug release behavior can be controlled by turning the core-sheath compositions, while retaining their bioactivity. Meanwhile, nanofibers exhibit much higher surface area-to- volume ratio which will facilitate the release the release of drugs and increase the contact area between cells and fibers, thereby enhancing drug uptake by cells^8–10^. Hence, electrospun nanofibers are potential candidates for nerve regeneration applications. Poly (lactic-co-glycolic acid) (PLGA) has been used in many tissue engineering application due to its biodegradability and biocompatibility^11–13^. Electrospun PLGA nanofibers have also been used to enhance the sciatic nerve regeneration in rat models^14^.

Bioactive molecule is another important factor in tissue engineering, which can improve neurogenesis in nerve injury site. However, most bioactive molecules such as growth factors and cytokines are expensive and instable in clinic efficacy, sometimes have side effects^15^. Plant extracts provide an alternative choice which always have multi-target and less side effects. And the plant extracts used for nerve repair should be effective in axonal regeneration and functional recovery. Wolfberry extracts is made up of a group of components including polysaccharides, betaine, zeaxanthin, beta-carotene, groups of vitamin, 19 kinds of amino acid and trace minerals^16^. It is believed that the polysaccharides of wolfberry, also named as Lycium barbarum polysaccharides (LBP), are the active components responsible for its various pharmacological properties including anti-aging, anti-tumor, anti-oxidation and immune modulation effects^17,18^. Increasing lines of evidence discloses the therapeutic effects of LBP on neuroprotection, which raises the possibility of using LBP as a neuroprotective agent after nerve injuries^17^. Studies involving primary cultured cortical neurons showed that LBP treatment reduced cellular death induced by amyloid-beta peptides and homocysteine insults^19–21^. Chan *et al*. reported that intragastric administration of LBP can reduce the death of retinal ganglion cells induced by experimental ocular hypertension^22^. Lau *et al*. found that LBP could promote the neuronal proliferation, differentiation of neural precursor cells^23^. Studies of Gao *et al*. also found that LBP could effectively protect PC12 cells and primary neurons against 6-hydroxydopamine-induced cell death^24^. Thus, introduction of bioactive chemicals such as LBP in neural tissue engineering may accelerate nerve regeneration. In our present study, we intended to investigate the effects of LBP along with PLGA nanofibrous scaffold on nerve cells behaviors such as proliferation, neuronal differentiation, neurite outgrowth and myelination *in vitro*. For the first time, we evaluated the effect of LBP on PC12 cells and Schwann cells proliferation and the differentiation of PC12 cells. In order to further confirm the potential role of LBP in peripheral nerve regeneration, core-shell structured PLGA-LBP nanofibers loading different dosage of LBP were fabricated via coaxial electrospinning. The topography of nanofibers was characterized by Scanning electron microscope (SEM), while the core-shell structure was confirmed by Transmission electron microscope (TEM) and fluorescent images. The surface hydrophilicity were evaluated by water contact angle. The mechanical properties of the scaffold were measured with tensile mechanical test. The *in vitro* release behavior of LBP from core-shell structured PLGA-LBP nanofibers was also determined. Then biocompatibility of the scaffold and the influence of scaffolds with diverse amount of LBP on nerve cells growth were tested in terms of proliferation and morphology of PC12 cells and Schwann cells. Furthermore, the neuronal differentiation of PC12 cells were performed on the scaffold in the absence and presence of Nerve growth factor (NGF). In order to explore the potential application of the PLGA-LBP nanofibrous scaffold in peripheral nerve repair, the neurite outgrowth of Dorsal root ganglion (DRG) neurons and the myelination of Schwann cell were further investigated.

## Results

**Figure 1:**
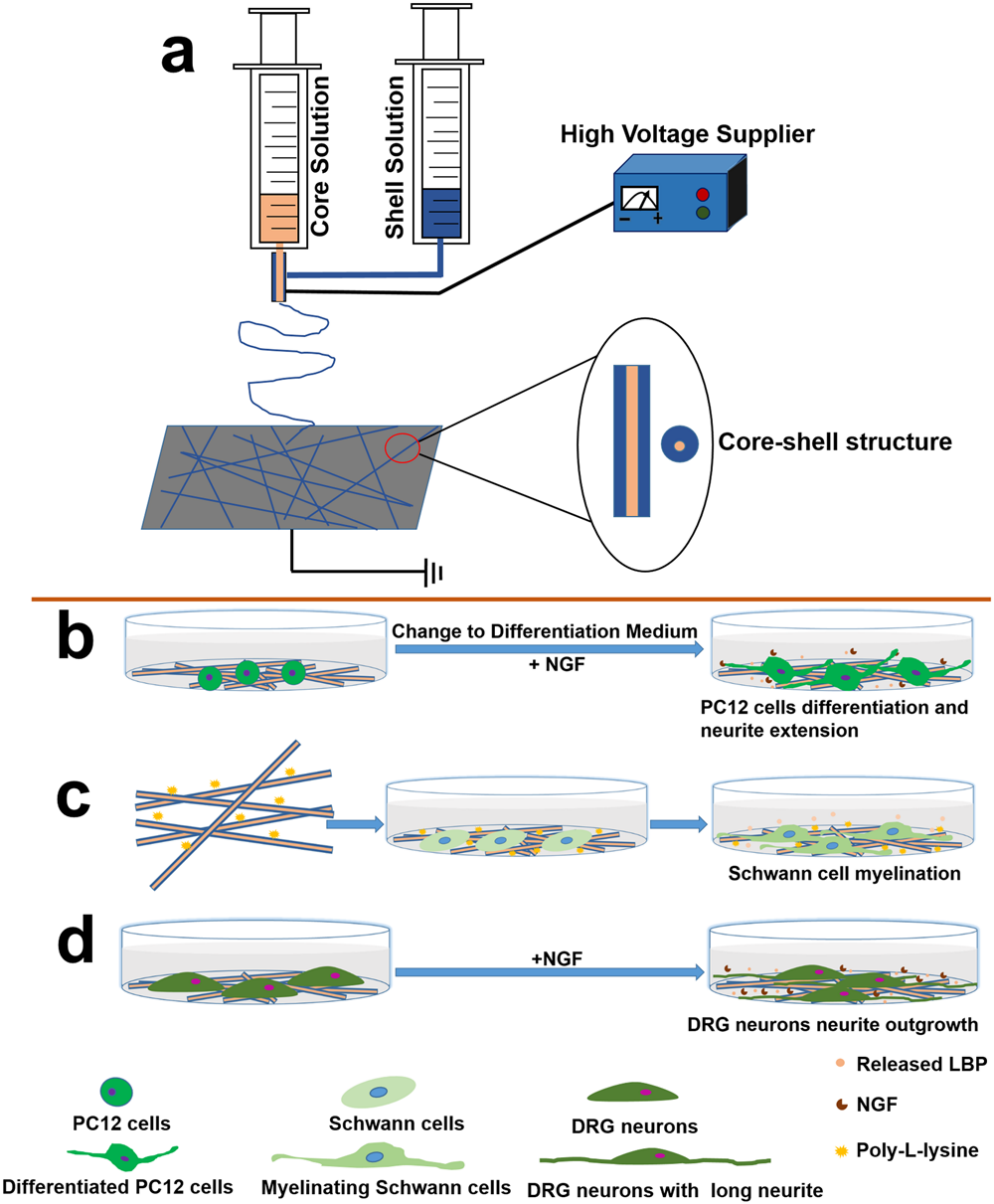
Schematic illustration of the preparation process and working hypothesis for coaxial electrospun fiber scaffolds. (**a**) The setup of coaxial electrospinning: the spinneret is composed of two concentric needles; the outer needle is used to deliver the shell solution (blue), while the inner needle is used to eject the core solution (Pink). (**b**) The LBP encapsulated in the core of coaxial fibers could be released in a sustained manner to promote differentiation of PC12 cells with Differentiation Medium^+NGF^. (**c**) Scaffolds coated with Poly-L-lysine could promote the adhesion of Schwann cells, and the released LBP could enhance the myelination of Schwann cells. (**d**) The neurite outgrowth of DRG neurons might be improved under the synergistic effect of LBP and NGF.

The objective of this research was to develop a coaxial electrospun nanofibrous scaffold containing LBP, which might improve both the differentiation of neurons and myelination of Schwann cells for peripheral nerve regeneration. The preparation process and working hypothesis of the scaffold are schematically illustrated in Fig. 1.

### Fabrication of PLGA-LBP nanofibrous scaffolds

Coaxial electrospinning was utilized to fabricate PLGA-LBP nanofibrous scaffold during this study. PLGA-LBP10, PLGA-LBP30, and PLGA-LBP50 were fabricated with LBP aqueous solution of three different concentration as the core solution. PLGA-LBP0 was fabricated with pure PLGA solution by conventional electrospinning. The fabrication process is schematically demonstrated in Fig. 1a.

### Physico-chemical properties of nanofibrous scaffolds

#### Surface morphology of nanofibrous scaffolds

**Figure 2:**
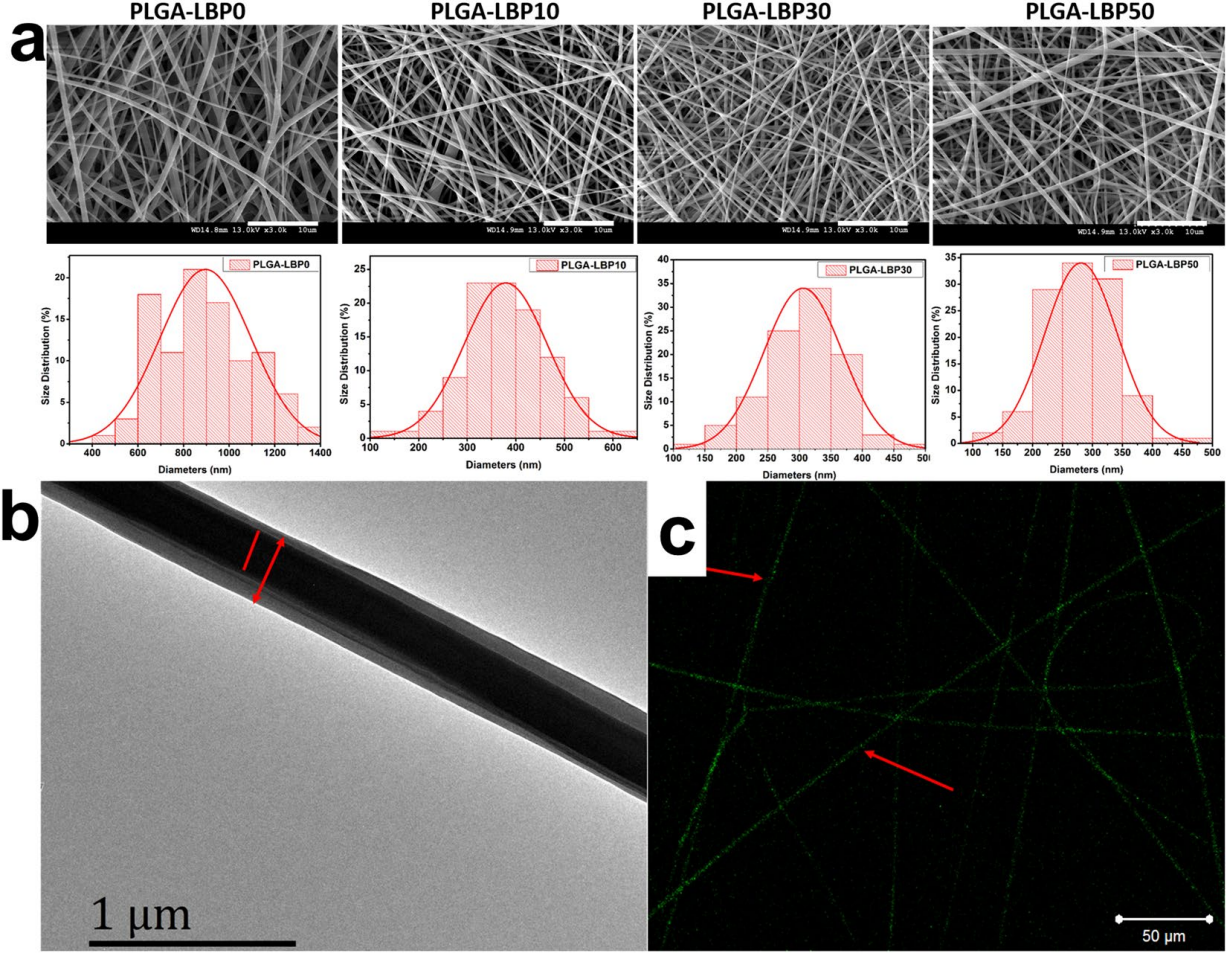
(**a**) SEM images of nanofibers and their diameter distributions. The scale bar is 10 μm; (**b**) TEM image of PLGA-LBP30 nanofibers; (**c**) Fluorescent image of PLGA-LBP30 nanofibers with FITC.

In nerve tissue, the topographical features of fibers play a significant role towards the behavior of cells. In Fig. 2a, the SEM images of the resultant nanofibers show the effects of various contents of LBP on nanofiber topographic features. It can be preliminary observed that all the nanofibers were randomly oriented, uniform and smooth in appearance without beads, indicating that LBP distributed uniformly in PLGA nanofibers. According to Fig. 2a and Table 1, the diameters of PLGA-LBP0, PLGA-LBP10, PLGA-LBP30 and PLGA-LBP50 were 896.35 ± 200.19 nm, 379.14 ± 85.39 nm, 305.88 ± 62.11 nm, and 295.90 ± 67.85 nm, indicating that incorporation of LBP caused great reduction in fiber diameters. Additionally, with the increase of LBP concentration, the average diameters of fibers decreased accordingly. The main reason is that the electric conductivity of electrospun suspensions increased with the increased content of LBP.

**Table 1:** Diameter, water contact angle and mechanical properties of scaffolds.

Scaffold	Diameter (nm/n = 100)	Water contact angle (°/n = 3)	Mechanical properties		
			Breaking tensile stress (MPa)	Breaking elongation (%)	Young’s Modulus (MPa)
PLGA-LBP0	896.35 ± 200.19	135.4 ± 4.4	5.06 ± 0.21	236.92 ± 69.70	200.79 ± 16.02
PLGA-LBP10	379.14 ± 85.39	135.8 ± 2.8	5.03 ± 0.52	26.73 ± 4.81	163.84 ± 11.08
PLGA-LBP30	305.88 ± 62.11	135.3 ± 4.1	4.22 ± 0.43	24.18 ± 5.92	153.03 ± 11.58
PLGA-LBP50	295.90 ± 67.85	135.8 ± 1.9	3.93 ± 0.39	18.97 ± 6.45	138.95 ± 10.18

#### Core-shell structure of nanofibrous scaffolds

The core-shell structure of LBP encapsulated nanofibers was confirmed by TEM and fluorescent images which were shown in Fig. 2b,c. The TEM image clearly indicated the formation of the core-shell structure which confirmed the incorporation of LBP within the polymeric shell. Fluorescent image showed the green light emitted from nanofibers duo to the existence of fluorescein isothiocyanate (FITC), suggesting that the mixture of LBP and FTIC was loaded inside the core of nanofibers successfully. The uniformly distributed green light along these fibers indicated the uniform dispersion of LBP along nanofiber axis.

#### The mechanical properties of nanofibrous scaffolds

Peripheral nerves are specific viscoelastic tissues with high mechanical properties^25^, so mechanical properties of nanofibers would play critical roles in peripheral nerve applications. The potential scaffolds should provide sufficient mechanical support for cells and axon regeneration. From Table 1 and Fig. S1, the tensile stress for PLGA-LBP0, PLGA-LBP10, PLGA-LBP30 and PLGA-LBP50 were 5.06 ± 0.21, 5.03 ± 0.52, 4.22 ± 0.43, 3.93 ± 0.39 MPa. Borschel *et al*.^26^ reported that stress of fresh rat sciatic nerve was 2.72 ± 0.97 MPa, therefore the PLGA-LBP nanofibers had sufficient tensile stress to be utilized as a nerve graft. As shown in Fig. S1, there was no significant difference between the tensile stress of scaffolds PLGA-LBP0 and PLGA-LBP10. However, the values of scaffolds PLGA-LBP30 and PLGA-LBP50 decreased gradually with the increasing content of LBP. As for the breaking elongations for different scaffolds, the values represented a great reduction from 236.92 ± 69.70% for PLGA-LBP0 to 18.97 ± 6.45% for PLGA-LBP50. The Young’s modulus decreased from 200.79 ± 16.02 MPa for PLGA-LBP0 to 138.95 ± 10.18 MPa for PLGA-LBP50, and there was a significant difference between scaffolds containing LBP and pure PLGA scaffolds. Compared to scaffolds PLGA-LBP10 and PLGA-LBP30, the decrease of Young’s modulus for PLGA-LBP50 was also statistically significant. The mechanical properties of nanofibrous scaffold decreased with the increased content of LBP, which was consistent with published results - the addition of low molecular drug had a “plasticizing” effect to fibers^27^. Even the mechanical properties decreased with the addition of LBP, the PLGA-LBP nanofibrous scaffolds were still suitable for nerve tissue engineering applications.

#### The surface hydrophilicity of nanofibrous scaffold

**Figure 3:**
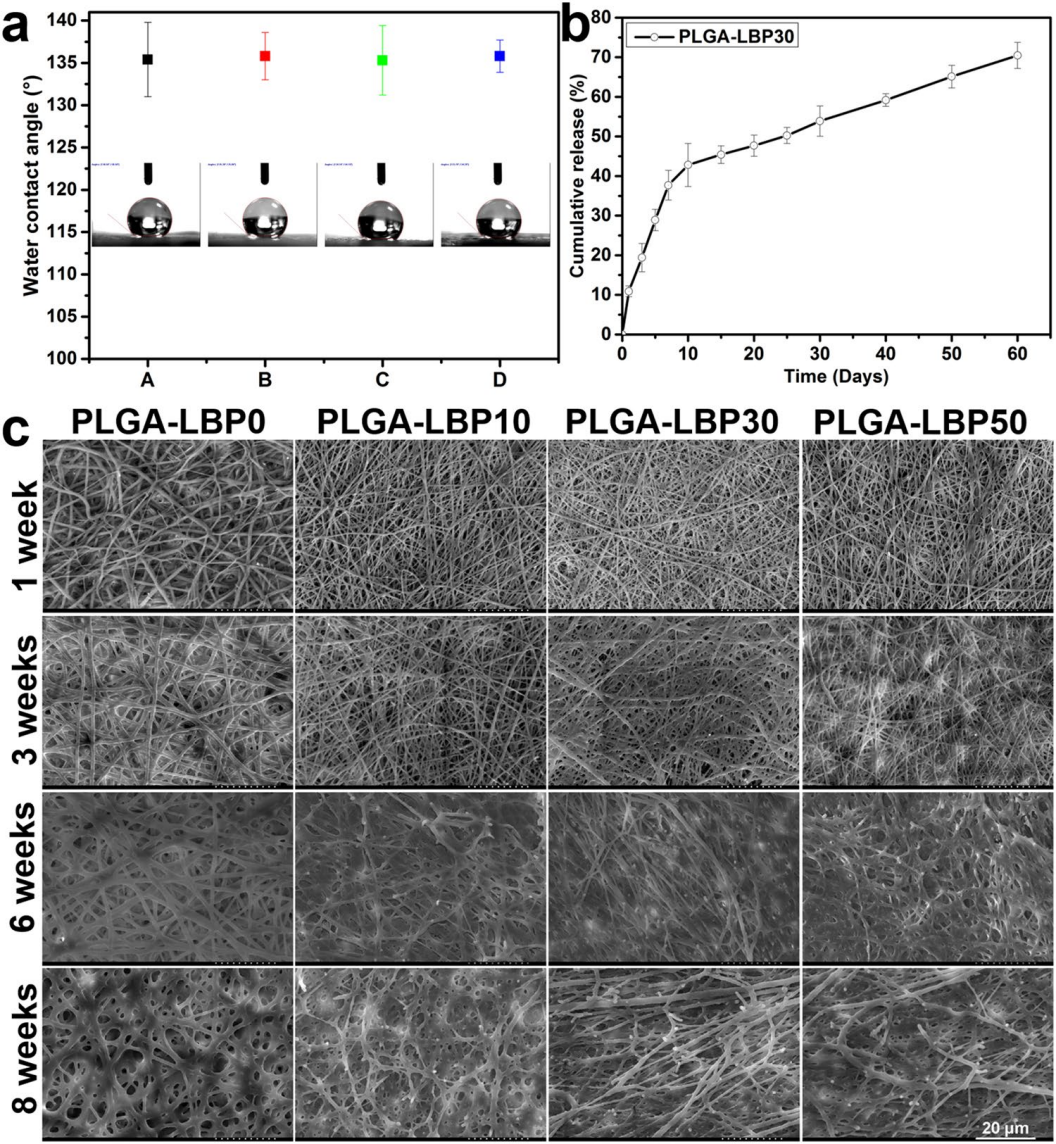
(**a**) Water contact angles of four different nanofibrous scaffolds. A: PLGA-LBP0 B: PLGA-LBP10 C: PLGA-LBP30 D: PLGA-LBP50. The data represent the mean of three independent experiments (n = 3, mean ± SD) (**b**) *In vitro* LBP release profile from PLGA-LBP30 nanofibrous scaffolds. The data represent the mean of three samples (n = 3, mean ± SD) (**c**) Typical SEM images of PLGA-LBP0, PLGA-LBP10, PLGA-LBP30 and PLGA-LBP50 nanofibrous scaffolds after degradation in PBS at 37 °C. The scale bar is 20 μm.

Water contact angle of nanofibrous scaffold reflects its surface hydrophilicity, which were shown in Table 1 and Fig. 3a. The water contact angles of PLGA-LBP0, PLGA-LBP10, PLGA-LBP30 and PLGA-LBP50 scaffolds were 135.4° ± 4.4°, 135.8 ± 2.8°, 135.3° ± 4.1° and 135.8° ± 1.9°. PLGA is a hydrophobic polymer, and the nanofibrous scaffold based on PLGA was also hydrophobic. LBP encapsulated inside the polymer matrix wouldn’t change the nanofibers surface hydrophilicity, so there was no obvious difference among these four different scaffolds in surface hydrophilicity.

### *In vitro* release behavior of LBP from core-shell nanofibers

The *in vitro* release behavior of drugs is affected by several factors including the degradation rate of the carriers, hydrophilicity of drugs and the drug distribution. In our study, coaxial electrospinning was used to fabricate core-shell structured nanofibers, which could encapsulate drugs in the core. The shell part could alleviate burst release and preserve the bioactivity of drugs as a barrier. Stimulating nerve growth at the injury site is most important during the first 10–15 days of repair^28^, and approximately 4 weeks were also very crucial for the peripheral nerve regenerations, which has been verified in rat sciatic nerve^29^. Hence, 4 weeks release of the LBP incorporated in the fibers would be favorable. The Ultra-violet absorption spectra of LBP solution with various concentration and the calculated standard line of LBP were shown in Figs S2 and S3, respectively. According to the accumulation release curve shown in Fig. 3b, the LBP exhibited a sustained release lasted for at least 2 months. The release kinetics can be described in two stages: a fast burst release (the first 7 days) followed by a sustained and constant release (the following 53 days). The initial fast release resulted from diffusion of LBP located near the surface of the fibers. The fast release for the first day was about 10%, which followed by a release until 7 days with a cumulative release of 40%. After 7 days, LBP displayed a sustained release over several weeks due to the increased diffusion path with the slow degradation of PLGA. During the period of 60 days of release, the cumulative release of LBP reached over 70%. In summary, the release results of LBP from PLGA-LBP30 nanofibrous scaffolds could satisfy the requirement in peripheral nerve regeneration.

### The *in vitro* degradation of nanofibrous scaffolds

The morphologies of PLGA, PLGA-LBP10, PLGA-LBP30 and PLGA-LBP50 nanofibrous scaffolds after 1, 3, 6, 8 weeks degradation were shown in Fig. 3c. All nanofibers remained the fibrous structures after 3 weeks degradation. After 6 weeks degradation, PLGA-LBP10, PLGA-LBP30 and PLGA-LBP50 started losing the fibrous structures, while the PLGA nanofibers still kept apparent fibrous structure with swollen appearance. After 8 weeks degradation, the fibrous structures of all scaffolds were blurred. The results indicated that the incorporation of LBP affected the degradation rate of scaffolds. With the release of LBP, more phosphate buffer solution (PBS) could enter inside the fiber and accelerated the degradation of scaffolds.

### The cell behavior of PC12 cells on scaffolds

#### The proliferation of PC12 cells

**Figure 4:**
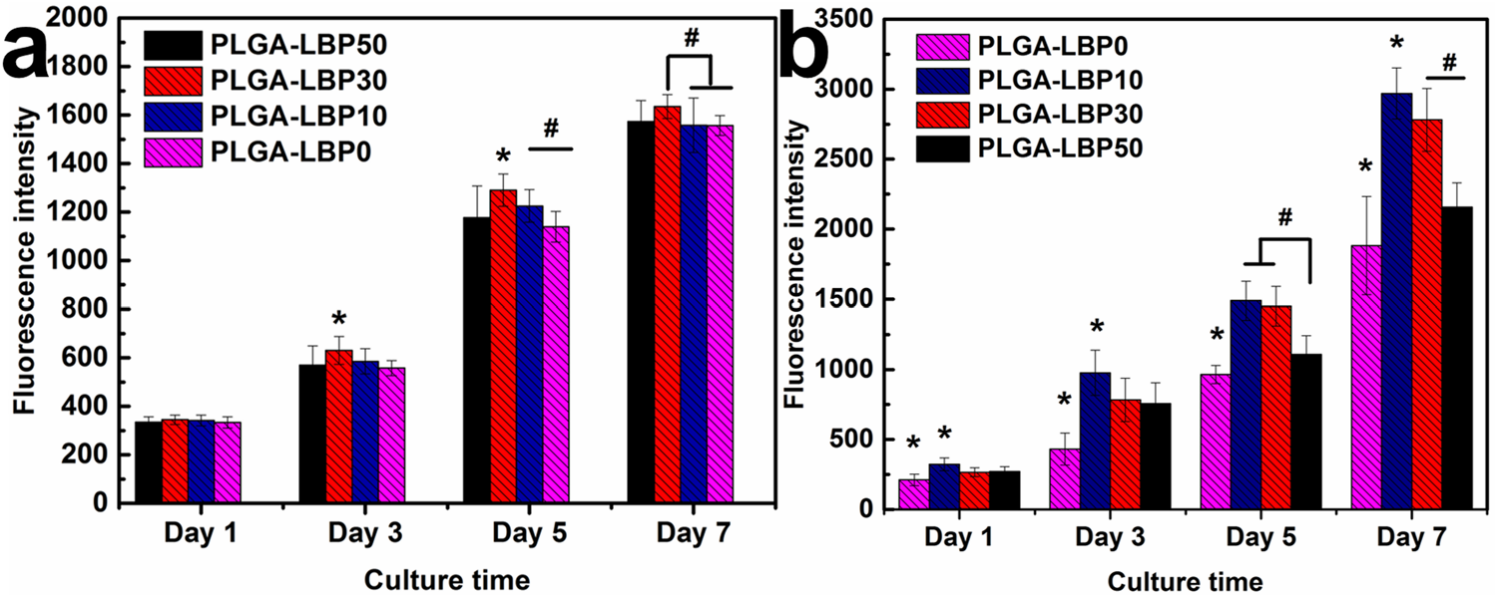
Proliferation of (**a**) PC12 cells and (**b**) Schwann cells on different scaffolds. The data represent the mean of three samples (n = 3, mean ± SD). *Denotes significance against all the other groups using one-way ANOVA test; ^#^denotes significance between two groups using one-way ANOVA test, P < 0.05.

In various studies, PC12 cells have been used as neuron precursors^30^. Therefore, the proliferation of PC12 cells should be considered in peripheral nerve tissue engineering. The ability of a tissue engineered scaffold to support the proliferation of cells is one of the major criteria to determine its application in target tissue regeneration. As shown in Fig. 4a, PC12 cells could attach, spread, and proliferate on all the nanofibrous scaffolds 1 day after seeding. However, the cell proliferation showed difference on different scaffolds on day 3, day 5 and day 7. PLGA-LBP30 nanofibrous scaffolds were more favorable for cell proliferation, which indicated that there was an optimal dosage for LBP used in peripheral nerve regeneration. Above all, the proliferation of PC12 was promoted by released LBP from nanofibrous scaffolds.

#### Neuronal differentiation of PC12 cells

**Figure 5:**
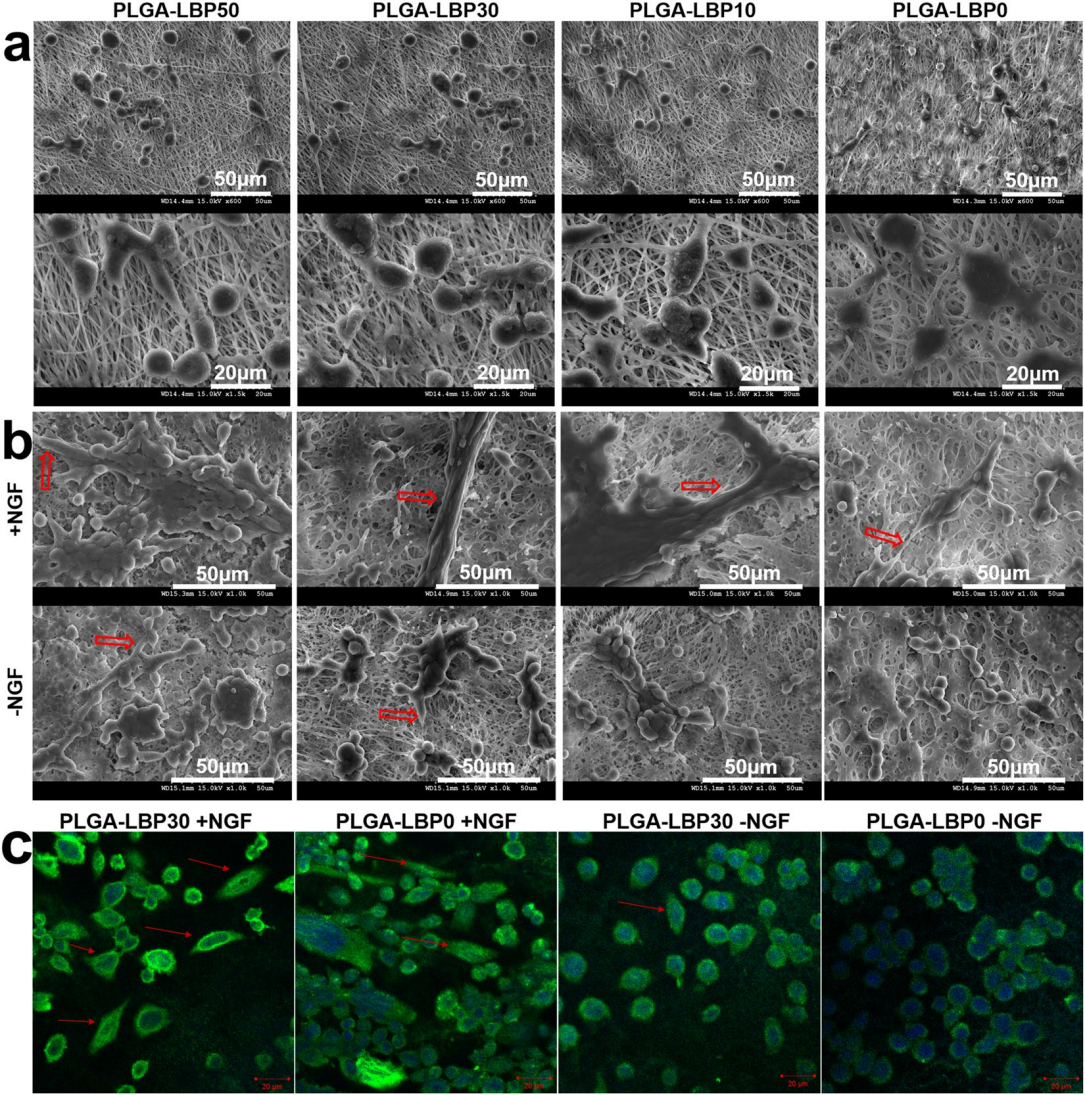
(**a**) Morphology of PC12 cells cultured with growth medium before differentiation; (**b**) Morphology of PC12 cells after differentiation on different nanofibrous scaffolds with different Differentiation Medium; (**c**) NF200 expression of PC12 cells on different nanofibrous scaffolds with different Differentiation Medium. The red scale bar is 20 μm. (+NGF: The cells cultured with Differentiation Medium with NGF; −NGF: The cells cultured with Differentiation Medium without NGF. The red arrow indicated the cell elongation).

PC12 cells are the most widely used cells for the neurogenesis study. The morphology changes of PC12 cells will reflect its differentiation directly. Figure 5a displayed the morphology of undifferentiated PC12 cells, which was smaller and spherical on all nanofibrous scaffolds. After being treated with Differentiation Medium with/without NGF for 10 days, the morphology of PC12 cell changed (Fig. 5b). A few cells grown on PLGA-LBP30 and PLGA-LBP50 nanofibrous scaffolds with Differentiation Medium^–NGF^ showed elongated shape, while on PLGA-LBP10 and PLGA-LBP30 nanofibrous scaffolds with Differentiation Medium^+NGF^, there were more cells showed a phenotype with elongated morphologies. Additionally, some cells on PLGA-LBP0 nanofibrous scaffolds with Differentiation Medium^+NGF^ also projected the neurites, while with Differentiation Medium^−NGF^, the cells were still showed undifferentiated phenotype. The phenotype of elongated morphologies confirmed the differentiation of PC12 cells. From all the aforementioned results, we concluded that NGF was essential in stimulating PC12 cells differentiation, but the stimulation of NGF could be enhanced with moderated amount of LBP.

Neurofilaments (NF) are the major groups of intermediated filaments which are found predominantly in cells and tissues of neuronal origin. Neurofilament proteins are synthesized in the neuronal perikarya, assembled to form filaments and then slowly transported within the axons towards the synaptic terminals^31^. Based on the results of PC12 cells proliferation and morphology of differentiated PC12 cells, we found that PLGA-LBP30 nanofibrous scaffolds should be more effective on nerve regeneration. Therefore, the expression of NF200 was evaluated to further confirm the neuronal differentiation of PC12 cells on PLGA-LBP30, and PLGA-LBP0 was used as control. The immunostaining of NF200 also enabled the comparison of cell phenotype and neurite outgrowth on different nanofibrous scaffolds. It can be seen from Fig. 5c that the results were consistent with morphology observation by SEM. The cells cultured on both scaffolds with Differentiation Medium with/without NGF all expressed NF200 protein, but the PLGA-LBP30 scaffolds showed more supportive for NF200 expression compared to cell differentiation observed on PLGA-LBP0 in Differentiation Medium with NGF. Most PC12 cells on PLGA-LBP30 nanofibrous scaffolds showed a neuro-like appearance with bigger soma and well grown neurites in Differentiation Medium with NGF. Meanwhile, PC12 cells on PLGA-LBP0 scaffolds in Differentiation Medium with NGF showed more neurites formed compared to that in Differentiation Medium without NGF. These results also indicated that NGF was an essential stimulation for PC12 differentiation, and the synergistic effect of LBP and NGF on neuronal differentiation of PC12 cells could be more effective.

### Cell behavior of Schwann cells and DRG neurons on scaffolds

#### The proliferation of Schwann cells

Schwann cells play a critical role in neuron survival and regeneration. They can provide neurotropic growth factors such as NGF and neurotrophin 3 (NT3) for neurite outgrowth as well as axonal nerve regeneration^32,33^. Additionally, the proliferation of Schwann cells can form columns of cells, known as brands of Büngner, which serve as a physical guide for regenerating nerve axons^34^. Therefore, the proliferation of Schwann cells should also be considered in peripheral nerve tissue engineering. According to Fig. 4b, the proliferation of Schwann cells showed difference after 1 day seeding. For all four time points, LBP contained nanofibrous scaffolds resulted in better cell proliferation, especially the PLGA-LBP10 nanofibrous scaffolds. The results suggested that the optimal dosage of LBP for Schwann cell proliferation was slightly lower than that for PC12 cells. Above all, the proliferation of Schwann cells was also promoted by released LBP from nanofibrous scaffolds.

#### The morphology study of Schwann cells

**Figure 6:**
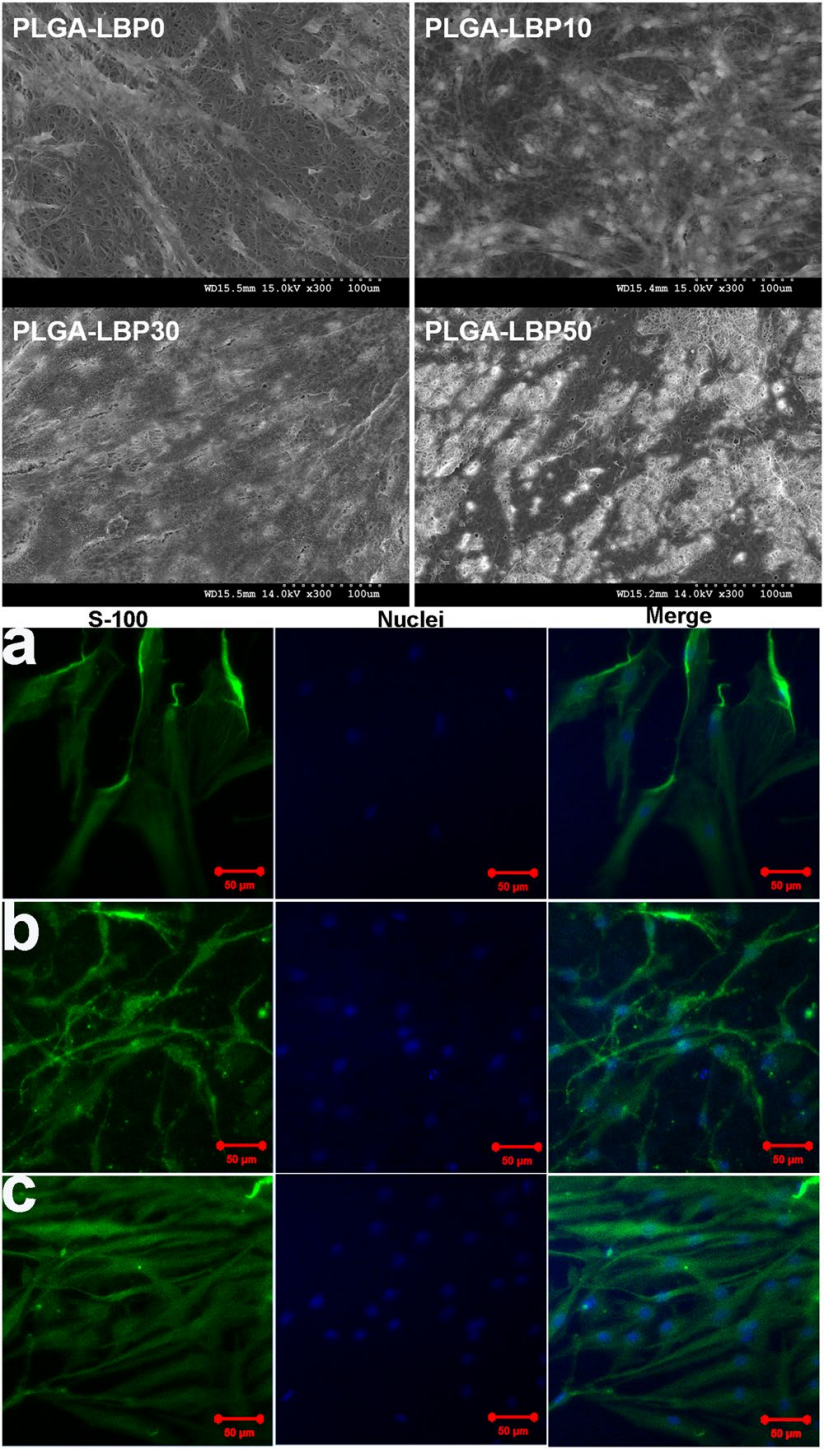
SEM images of Schwann cells on different nanofibrous scaffolds and confocal micrographs of Schwann cells on (**a**) TCP, (**b**) PLGA-LBP0 and (**c**) PLGA-LBP30 nanofibrous scaffolds after 7 days of *in vitro* culture.

It has been known that Schwann cells play a critical role via the synergetic effect with neurons and macrophages during the regeneration process of injured PNS^35–37^. Thus to further confirm the interactions of Schwann cells with electrospun nanofibrous scaffolds, the morphology and phenotype of cells were evaluated after 7 days *in vitro* culture by SEM and immunostaining. The SEM images in Fig. 6 demonstrate the interaction of Schwann cells with four different nanofibrous scaffolds. The cells interacted and integrated well with all scaffolds, and Schwann cells exhibited a typical spindle-like shape with bio-polar extensions. Notably on LBP loaded nanofibrous scaffolds, Schwann cells grew in higher density, and the interaction between cell-cell and cell-scaffold was much better than pure PLGA scaffolds, especially on PLGA-LBP30 scaffolds. In all, the growth of Schwann cells was enhanced with LBP released from the scaffolds, and the PLGA-LBP30 scaffolds was the best choice for application in nerve regeneration.

In order to corroborate the results obtained by SEM observation, immunostaining with S-100 antibody, which was a specific protein for Schwann cells was conducted. According to the aforementioned results, PLGA-LBP30 and PLGA-LBP0 scaffolds were used for this evaluation, and tissue culture plate (TCP) was chosen as control. Confocal images presented in Fig. 6 confirmed that the cells showed a higher proliferation on PLGA-LBP30 scaffolds. Compared to cells on TCP, the Schwann cells exhibited higher maturation on both nanofibrous scaffolds especially on PLGA-LBP30.

All these results indicate that the PLGA-LBP30 scaffolds were the most favorable for both PC12 and Schwann cells growth. Therefore PLGA-LBP30 scaffold was selected for further study, and PLGA-LBP0 scaffold was used as control.

#### Myelin basic protein (MBP) expression of Schwann cells

**Figure 7:**
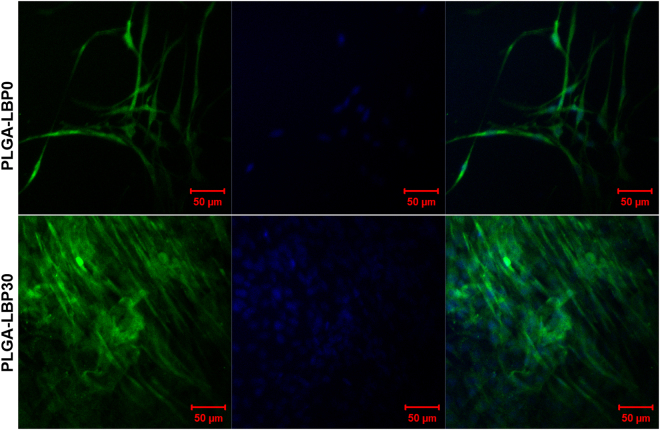
MBP expression of Schwann cells. Immunofluorescence images of Schwann cells cultured on PLGA-LBP0 and PLGA-LBP30 scaffolds for 10 days (Green: MBP; Blue: Nuclei stained with DAPI).

Myelination of Schwann cells is critical for the success of peripheral nerve regeneration. The matured myelinating Schwann cells are able to secrete various neurotrophins such as NGF, brain derived neurotrophic factor (BDNF), and ciliary neurotrophic factor (CNTF), which can promote the neurogenesis and guide axon elongation^38,39^. To investigate the myelinating of Schwann cells, the expression of marker of Schwann cells myelination, such as myelin basic protein (MBP) was measured with immunostaining. As shown in Fig. 7, the cells on both scaffolds expressed MBP, but obviously stronger fluorescence was observed in cells on PLGA-LBP30 scaffolds. Schwann cells showed a mature bipolar shape and arranged in bundles in PLGA-LBP30 scaffolds. As we know, the Schwann cells usually migrate and form a column of cells known as brands of Bünger to guide the regeneration of axon after nerve injuries. So PLGA-LBP30 nanofibrous scaffolds have the potential to enhance the myelination of Schwann cells in peripheral nerve regeneration.

#### Neurite outgrowth of DRG neurons on scaffolds

**Figure 8:**
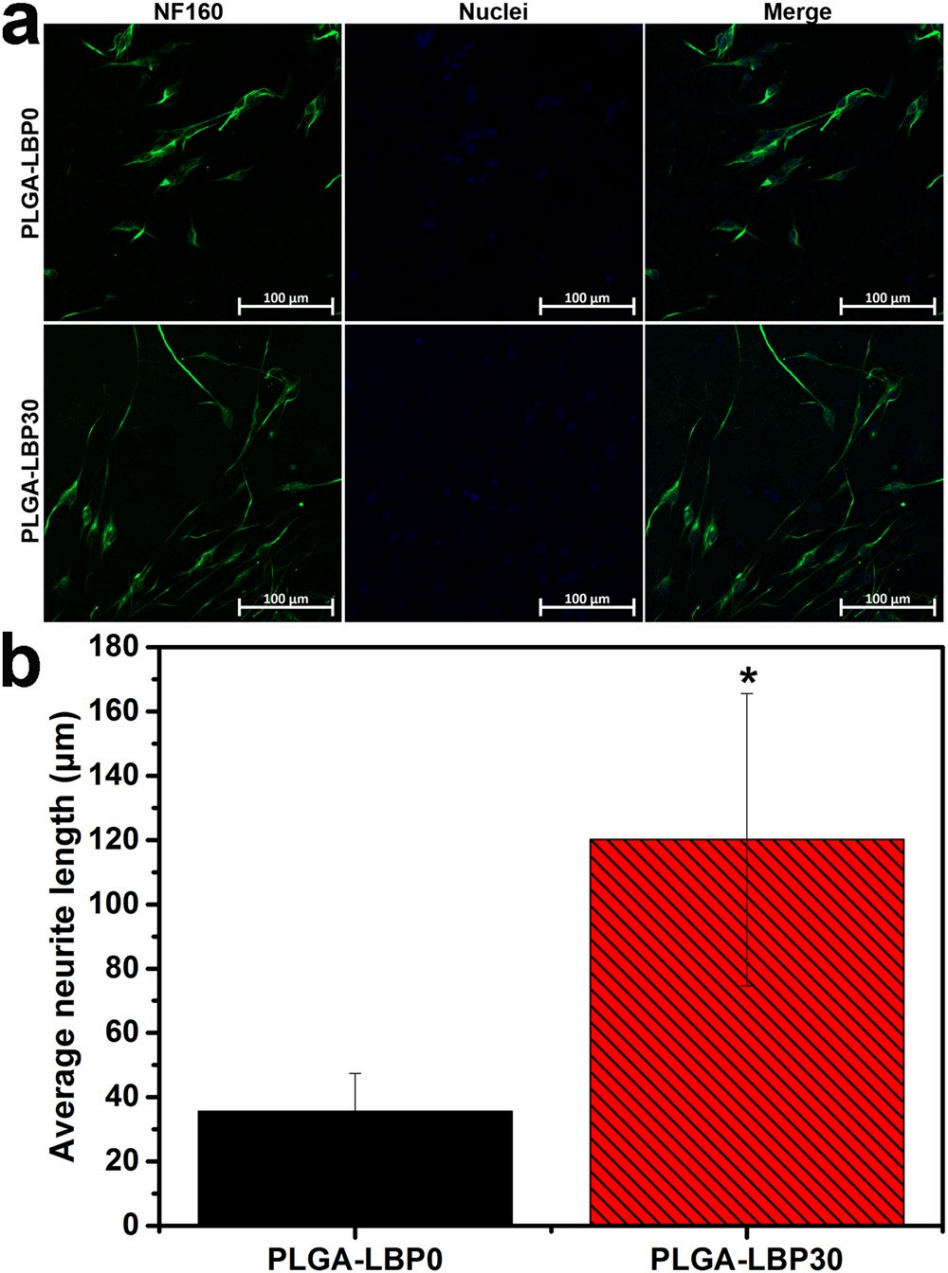
(**a**) Fluorescence images of DRG neurons cultured on PLGA-LBP0 and PLGA-LBP30 scaffolds for 7 days (Green: NF160 stained with Alexa Fluor 488-conjugated goat anti-mouse lgG H&L; Blue: nuclei counterstained with DAPI). (**b**) Neurite length of DRG neurons on PLGA-LBP0 and PLGA-LBP30 scaffolds after 7 days culture (Data were mean ± SD, n = 10; *denotes significance between two groups using one-way ANOVA test; P < 0.05).

We have demonstrated that LBP loaded PLGA nanofibrous scaffolds promoted proliferation and neurite outgrowth of PC12 cells. For further study, we performed evaluation of neurite outgrowth behavior of the rat primary DRG neurons. The expression of Neurofilament 160 protein (NF160) was evaluated by immunostaining and observed by LSCM. The results in Fig. 8a revealed that NF160 expression was significantly improved in the PLGA-LBP30 scaffolds. We also found that the neurite outgrowth of the DRG neurons on PLGA-LBP30 scaffolds was faster than that on PLGA-LBP0 scaffolds. To determine whether the LBP encapsulation in the scaffolds influenced the extent of neurite growth or not, the lengths of all of the representative neurites were measured using ImageJ software (Fig. 8b). The neurite length is defined as the linear distance from the tip of the neurite to the cell junction. The average neurite length of DRG neurons on PLGA-LBP0 scaffolds was 35.62 ± 11.83 μm, while the value increased to 120.17 ± 45.44 μm for DRG neurons cultured on PLGA-LBP30 scaffolds. Moreover, the longest neurite was up to 251.51 μm on PLGA-LBP30 scaffolds. Comparison of the mean neurite lengths of the DRG neurons cultured on PLGA-LBP30 scaffolds with those of neurons cultured on PLGA-LBP0 scaffolds revealed that the axon elongation was significantly enhanced on PLGA-LBP30 scaffolds. This may be attributed to the released LBP from the scaffolds.

## Discussion

Wolfberry has been used as functional food as well as medicinal herbs in Asian countries for more than thousands of years. LBP as the active component extracted from Wolfberry has been shown to be neuroprotective in many studies^17^. Electrospun nanofibers have been widely used as scaffold for peripheral nerve tissue engineering application. A variety of natural and synthetic polymers have been fabricated into nanofibers to support neural tissue engineering development. PLGA, as a biocompatible and biodegradable polymer material, has been widely used in peripheral tissue engineering^15,40^. The present study was intended to fabricate PLGA nanofibers loaded with LBP via coaxial electrospinning. Thus, a synergistic effect was produced by the combination of nerve tissue engineering and drug therapy to accelerate the nerve regeneration.

In the present study, four different groups of nanofibers were prepared according to the different concentration of LBP. The diameters of fibers gradually decreased from about 900 nm to about 300 nm with the increase of LBP concentration (Fig. 2). Early studies have reported that randomly oriented nanofibers with smaller diameters (~300 nm) are more favorable for nerve cells growth compared to nanofibers with large diameters (~750 nm)^41^. The LBP was indeed encapsulated within the polymer shell and could be released slowly from the fibrous scaffold (Fig. 3b). The feasibility of LBP encapsulated nanofibers for peripheral nerve tissue engineering application was evaluated by identification of nerve cell behaviors on different nanofibers. PC12 cells are derived obtained from a pheochromocytoma of the rat adrenal medulla, which are widely used as model cells in neurophysiological and neuropharmacological studies^42^. Schwann cells are the glial cells in peripheral nerve system, which play a key role in Wallerian degeneration and subsequent regeneration, and provide a suitable environment for regeneration. In this study, the proliferation of both PC12 and Schwann cells were significantly enhanced in LBP containing nanofibers, especially on PLGA-LBP30 group (Fig. 4). Yu *et al*. found that LBP could prevent neuronal death via both necrosis and apoptosis with a wide range of effective dosages *in vitro* by inhibiting two key pro-apoptotic signaling pathways which were c-jun N-terminal kinase (JNK) and double-stranded-RNA-dependent protein kinase (PKR)^19,20^. Additionally, Ho *et al*. reported that cell death of Rat primary cultured cortical neurons treated with Aβ_25–35_ and Aβ_1–42_ was reduced by LBP through the improve of phosphorylation of Akt^21^. Consequently, the application of LBP loaded nanofibrous scaffolds in nerve injured sites may inhibit the apoptosis of neurons. The differentiation of PC12 cells induced by NGF was also improved on LBP loaded nanofibers, as evidenced by morphological evaluation and immunofluorescence staining (Fig. 5). Although the mechanism of LBP in PC12 cells differentiation was not known exactly, our results revealed that the differentiation of PC12 cells induced by NGF could be promoted by LBP to a certain extent. Previous studies by Ho *et al*. reported that LBP could reduce the cell death of Rat primary cultured cortical neurons treated with homocysteine or glutamate, and the possible mechanism was to up regulating the phosphorylation of Extracellular Regulated Kinase (ERK)^21,43^. It has been known that the process of phosphorylation of ERK is the necessary requirement of PC12 cells differentiation induced by NGF^44–46^. Thus, we supposed that the LBP could facilitate the PC12 cells differentiation induced by NGF through the up regulation of phosphorylation of ERK. Additionally, the present study found that the expression of MBP in Schwann cells showed significant enhancement on PLGA-LBP30 group (Fig. 7). Gao *et al*. reported^24^ that LBP could decrease the level of intracellular Ca^2+^, while some research have shown that intracellular Ca^2+^ level appears to affect cell viability, proliferation and differentiation in Schwann cells and neuronal cells^47–49^. Therefore, we considered that the Schwann cells myelination was improved by LBP through the decrease of intracellular Ca^2+^. Then the decrease of intracellular Ca^2+^ level may lead to inactivation of protein kinase C (PKC), which is a key protein kinase enzyme involved in other signal pathways such as mitogen-activated protein kinase (MAPK) to indirectly influence Schwann cells myelination^50,51^. Rat DRG neurons showed better viability and neurite out growth on LBP containing nanofibers, which could be seen in Fig. 8. We surmised that the synergistic effect of NGF and LBP was more effective than the independent induction of NGF in promoting the axon growth. In brief, the existence of LBP showed a remarkable increase in the neurite outgrowth of the rat DRG neurons and up-regulation of the NF160 expression, although the synergistic reaction mechanism between NGF and LBP was not known completely.

Taken together, these results demonstrated that LBP has a positive effect on nerve cells proliferation and differentiation *in vitro*. There was a beneficial synergistic effect between NGF and LBP in promoting PC12 cells differentiation and axonal extension of neurons. To allow regeneration following peripheral nerve injury, the microenvironment must be permissive enough for neurite outgrowth. Therefore, the LBP loaded nanofibrous scaffolds, especially PLGA-LBP30, could be a promising substrate for target peripheral nerve injury repair.

## Materials and Methods

### Materials

The 75/25 Poly (lactic-co-glycolic acid) (PLGA) (inherent viscosity 0.61 dL/g) was purchased from Jinan Daigang Biomaterial CO., Ltd, China. Lycium barbarum polysaccharide was provided by Guangdong-Hongkong-Macau Institute of CNS Regeneration (GHMICR), Jinan University. 1,1,1,3,3,3-Hexafluoro-2-propanol (HFP), glutaraldehyde, Dulbecco’s modified eagle’s medium (DMEM/F12), were purchased from Sigma, Singapore. Nerve growth factor (NGF) was purchased from Millipore, Singapore. Rat pheochromocytoma (PC12) cells in the adherent type and Schwann cells were obtained from ATCC, USA, while fetal bovine serum (FBS), horse serum (HS) and trypsin/EDTA were purchased from GIBCO Invitrogen, USA. Alamar Blue (AbD Serotec) was purchased from Chemoscience, Singapore. Schwann cell medium was purchase from Gene-Ethics Asia Pte Ltd, Singapore.

### Fabrication of scaffolds

28% w/v PLGA solution was prepared by dissolving PLGA into HFP followed by a magnetic stirring at room temperature overnight. Meanwhile, LBP aqueous solution with 10 mg/ml, 30 mg/ml, and 50 mg/ml concentration were prepared separately. Core-shell structured PLGA-LBP nanofibers were fabricated via our lab’s electrospinning set-up as shown in Fig. 1a. The PLGA solution and LBP aqueous solution were separately placed into 5 ml plastic syringes and fed through a compound nozzle with an inner needle coaxially placed inside an outer one at a rate of 1 ml/h and 0.2 ml/h using two syringe pump respectively. The exit orifice diameters of the inner and outer needles were 0.5 and 0.8 mm. The high voltage applied during electrospinning was 18 kV, while the distance between the tip of the needle and grounded steel plate covered with aluminum foil was set at 13 cm. The nanofibrous scaffolds fabricated with LBP aqueous solution of 10 mg/ml, 30 mg/ml, and 50 mg/ml concentration were separately referred as PLGA-LBP10, PLGA-LBP30, and PLGA-LBP50. Pure PLGA scaffolds were also prepared as control by conventional electrospinning, and labeled as PLGA-LBP0.

### Characterization of scaffolds

#### The surface morphology of nanofibrous scaffolds

The scanning electron microscope (SEM) was applied to observe the surface topography of fabricated nanofibrous scaffolds. The electrospun nanofibers were sputter-coated with gold (JEOL JFC-1200 Fine Coater, Tokyo, Japan) and then visualized using a SEM (Model S-4300, Hitachi, Tokyo, Japan). The average diameter of the nanofibers was calculated from 100 random points chosen from the SEM images with analysis by Image J software (National Institute of Health, Bethesda, MD).

#### The core-shell structure of nanofibrous scaffolds

The core-shell structure of the nanofibers was verified using transmission electronic microscopy (JEM-3010, JEOL, Tokyo, Japan) at 200 kV. The samples for TEM observation were prepared by collecting nanofibers with carbon-coated copper grids. Laser scanning confocal microscopy (LSCM, Zeiss LSM700) was used to further confirm the distribution of LBP in the core of nanofibers, and to do so, the LBP aqueous solution used for electrospinning was mixed with FITC.

#### The mechanical property of nanofibrous scaffolds

Tensile properties of the nanofibrous scaffolds were evaluated using a universal materials tester (H5K-S, Hounsfield, UK) at an ambient temperature of 20 °C and humidity of 65%. Tests were performed on rectangular specimens with dimensions of 30 × 10 mm. A cross-head speed of 10 mm min^−1^ was applied for all specimens during the process. Six specimens of each sample were measured.

#### The measurement of water contact angle

Hydrophilicity of the electrospun nanofibrous scaffolds was measured by water contact angle measurement using VCA Optima Surface Analysis System (AST products, Billerica, MA, USA). To prepare samples for water contact angle measurement, the nanofibers were collected on coverslips and placed on the testing plate. Then deionized water was dropped on the specimens and the data was recorded. Three different points of the same specimen were measured and each sample was performed in triplicate.

### Release of LBP from core-shell nanofibers *in vitro*

PLGA-LBP30 nanofibrous scaffold was used to evaluate the release behavior of LBP *in vitro*. 10 mg of PLGA-LBP30 nanofibrous scaffolds were soaked in a 2 ml centrifuge tube with 1 ml of PBS. Subsequently, the centrifuge tubes with nanofibrous mats were all incubated in a continuous horizontal shaker at a speed of 150 rpm at 37 °C. At preset time points, the PBS was retrieved completely from the tubes and the equal volume of fresh PBS was replaced for continuous incubation. The LBP concentration was determined by UV-vis spectrophotometer at wavelength at 260 nm. The cumulative amount of released LBP was calculated. The samples for each time point were run in triplicate.

### Degradation of nanofibrous scaffolds *in vitro*

The degradation of nanofibrous scaffolds were performed as described previously^52^. Briefly, the nanofiber meshes were cut into small square pieces. Then the specimens were immersed in 10 ml of PBS in 15 ml centrifuge tubes. Subsequently, the tubes were kept in a shaking incubator at 37 °C operating at 150 rpm. At each particular time point of study, the specimens were taken out from the tubes, rinsed several times with distilled water to remove residual buffer salts, and dried in a vacuum desiccator. The morphology was observed with SEM as described previously.

### Cell culture *in vitro*

#### PC12 cell culture

PC12 cells were cultured in normal Growth Medium composed of Dulbecco’s modified eagle’s medium (DMEM/F12) supplemented with 10% horse serum (HS), 5% fetal bovine serum (FBS), and 1% antibiotic/antimycotic solution in a 75 cm^2^ cell culture flask. Cells were maintained at 37 °C in a humidified incubator with 5% CO_2_, and the culture medium was changed once in every two days. The nanofibrous scaffolds for cell culture were collected with 15 mm cover slips. All the samples were sterilized for 5 h under UV light in biological safety cabinet (Bioclassic Class II Series Type A2, Gelman Laminar Airflow Systems) and then placed in a 24-well plate pressed with stainless steel rings. Subsequently all samples were washed thrice with PBS, and immersed in Growth Medium until cell seeded. After the confluence was sufficient, PC12 cells in flask were detached by trypsin/EDTA, counted by automated cell counter, and seeded on all nanofibrous scaffolds with a density of 1.0 × 10^4^ cells/well.

#### Schwann cell culture

Similarly, the Rat Schwann cells were grown in SCM medium with 10% FBS. And before seeding the cells, the scaffolds need coating with Poly-L-lysine. In detail, the scaffolds were washed with PBS for three times after sterilization under UV light, and then incubated with 200 μl Poly-L-lysine solution (Sigma, Singapore) for 2 h in 37 °C incubator. Subsequently, the scaffolds were washed again with PBS for three times, and placed in an incubator overnight to allow the scaffolds to be dry fully. All the other followed process was the same as PC12 cells.

### Cell behavior of PC12 cells on scaffolds

#### The proliferation of PC12 cells

The proliferation of PC12 on different scaffolds was performed after 1, 3, 5, 7 days of culture by means of the Alamar Blue assay. At predetermined time points, medium was carefully removed and 1 ml of Alamar Blue solution (10% Alamar Blue, 90% DMED/F12 medium) was added to each well followed by incubation in darkness for 4 h under 37 °C. After incubation, the solution was pipetted from each well into 96-well plate and read at 560 nm (excitation)/590 nm (emission) by a Varioskan flash reader (Thermo Fisher Scientific, MA, USA).

#### Neuronal differentiation of PC12 cells

For differentiation study of PC12 cells, the medium was changed from Growth Medium to Differentiation Medium^+NGF^ composed of DMED/F12 supplemented with 1% HS, 0.5% FBS, 1% antibiotic/antimitotic solution and 100 ng ml^−1^ NGF after cells seeded for 1 day. For comparison, cells were also cultured with the same medium above without NGF which is referred as Differentiation Medium^−NGF^.

The morphology of differentiated PC12 cells was different from its original ones. To evaluate the neuronal differentiation of PC12 cells, the morphological changes were observed with SEM after 10 days of incubation in Differentiation Medium. Meanwhile, the morphology of cells cultured on scaffolds with Growth Medium was also observed as control. After 10 days incubation, cells-scaffolds constructs were rinsed twice with PBS and then fixed with formalin for 20 min. After that, all samples were rinsed thrice with PBS and dehydrated with a series of graded ethanol (50%, 70%, 90% and 100%) for 15 min each, followed by air-drying overnight in room temperature. Finally, the dry samples were sputter coated with gold and observed under SEM.

The neuronal differentiation of PC12 cells was also evaluated by immunostaining of neuronal specific protein-neurofilament 200 (NF200). After 10 days of cell culture in Differentiation Medium, all scaffolds were rinsed with PBS, fixed in formalin for 20 min and permeabilized with 0.1% Triton-X100 for 5 min. Then 3% BSA solution was used to block nonspecific binding for 90 min. After that, the samples were stained with primary antibody, anti-NF200 produced in rabbit (Sigma) at a dilution of 1:100 for 2 h at room temperature. Subsequently, all samples were washed thrice with PBS and stained with secondary antibody, FITC conjugated goat anti rabbit (Sigma) at a dilution of 1:300 for 1 h. After rinsing with PBS for five times, the scaffolds were treated with mounting medium with DAPI (Vector Laboratories, USA). Finally, the immunostained samples were visualized under LSCM to observe the cell phenotype and neurite extension of differentiated PC12 cells.

### Cell behavior of Schwann cells and DRG neurons on scaffolds

#### The proliferation of Schwann cells

The proliferation of Schwann cells on different scaffolds was performed after 1, 3, 5, 7 days of culture by means of the Alamar Blue assay. The detailed process was the same as what described in section of *the proliferation of PC12 cells*.

#### Cell morphology study of Schwann cells

The morphology of Schwann cells on different scaffolds was observed by both SEM and fluorescent microscopy. For SEM observation, all the process was the same as what described in section of *Neuronal differentiation of PC12 cells*. For the fluorescent microscopy observation, immunostaining of specific protein for Schwann cells, namely S100, was carried out. And the followed process was similar with that described in section of *Neuronal differentiation of PC12 cells*. The primary antibody used in this study was rabbit anti-S100 (diluted at 1:200, Sigma).

#### Myelin basic protein (MBP) expression of Schwann cells

Myelination of Schwann cells is critical for the success of peripheral nerve regeneration. To evaluate the influence of PLGA-LBP nanofibrous scaffold on modulating myelination of Schwann cells, the expression of MBP of Schwann cell was determined with immunostaining. The process of immunostaining was the same as what described in section of *Neuronal differentiation of PC12 cells*. Anti-MBP antibody produced in rabbit (diluted at 1:200, Sigma) was used as the primary antibody in this study.

#### DRG neurons outgrowth on nanofibrous scaffolds

DRG neurons were isolated from the embryo of the 14 day pregnant rat. Before seeding the DRG neurons, the samples were exposed to UV light overnight for sterilization, followed by thrice rinse with PBS. The culture medium for DRG neurons was Neuralbasal medium with 1% Penicillin-Streptomycin Solution, 2% B27 supplement factors and 20 ng ml^−1^ NGF. The culture medium with NGF was replaced every two days. After 7 days incubation, the DRG neurons were fixed for 30 min in a 4% paraformaldehyde at 4 °C and then washed three times with PBS. Subsequently, the DRGs were immunostained with the primary antibody solution overnight at 4 °C. After washing with PBS for three times, the samples were incubated with Goat anti-rabbit IgG Alexa 488 (Invitrogen, Camarillo, CA, USA) diluted 1:200 in PBS as the secondary antibody. The nuclei were stained with DAPI for 15 min finally (Thermo Scientific). The outgrowth of immunostained DRG neurons were visualized under LSCM.

### Statistical analysis

Statistical analyses were carried out using one-way ANOVA followed by Tukey’s test. All data were reported as mean ± standard deviation (SD). Differences were considered statistically significant when P < 0.05.

## Electronic supplementary material

Supplementary Information
