# Supplementary material for: Lycium barbarum polysaccharide encapsulated Poly lactic-co-glycolic acid Nanofibers: cost effective herbal medicine for potential application in peripheral nerve tissue engineering
Source: Sci Rep. 2018 Jun 6;8:8669. doi: 10.1038/s41598-018-26837-z (PMC5989206; doi:10.1038/s41598-018-26837-z)
Supplement: Supplementary file 1 — Supplementary Information [file 41598_2018_26837_MOESM1_ESM.docx]

Lycium barbarum polysaccharide encapsulated Poly lactic-co-glycolic acid Nanofibers: cost effective herbal medicine for potential application in peripheral nerve tissue engineering

Jing Wang^1, 2^, Lingling Tian^2^, Liumin He^3, 5^, Nuan Chen^2^, Seeram Ramakrishna^2, 4^, Kwok-Fai So^*, 4, 5^, Xiumei Mo^*, 1^


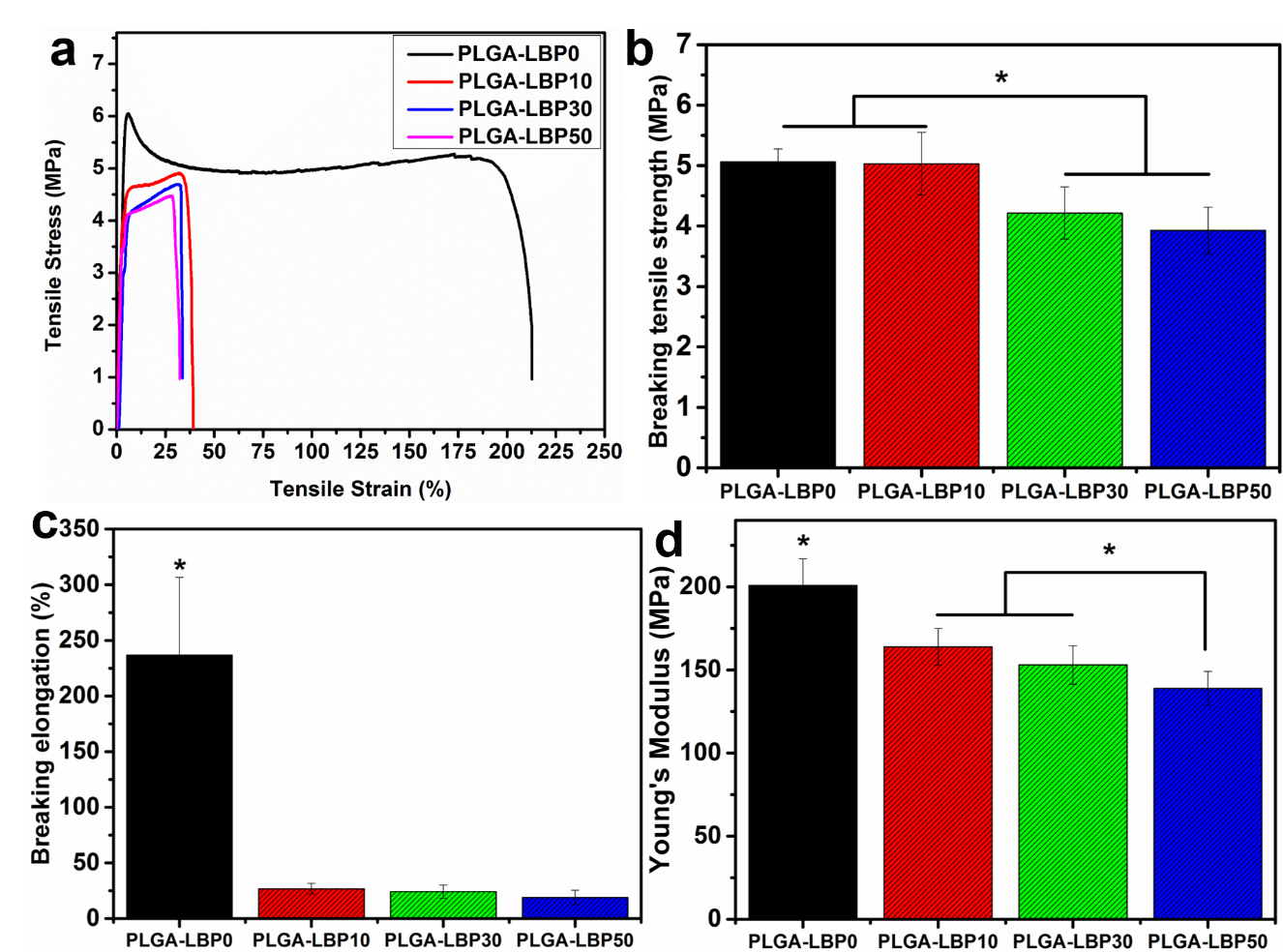


**Figure S1.** Mechanical properties of four different nanofibrous scaffolds, (a) The representative tensile stress-strain curves; (b) breaking stress; (c) breaking elongation and (d) Young’s modulus. The data represent the mean of six samples (n = 6, mean ± SD); * refer to the significant difference using one –way ANOVA test, P < 0.05.





**Figure S2.** Ultra-violet absorption spectra of LBP solution with various concentration.


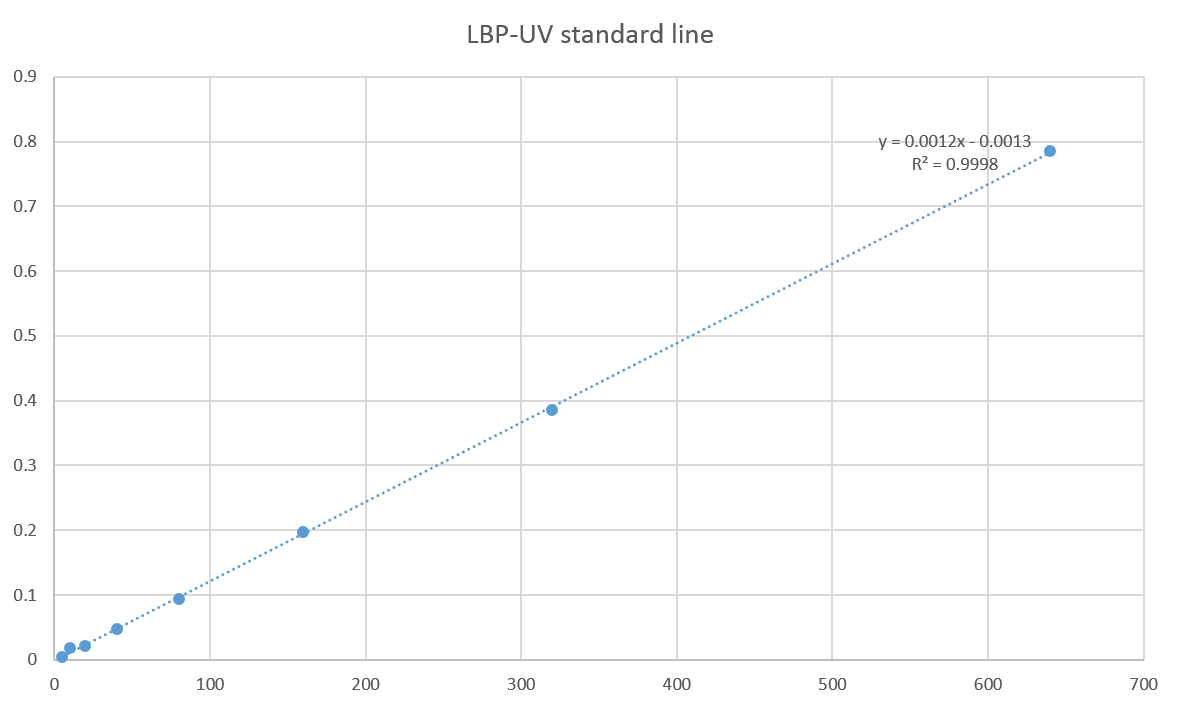


**Figure S3.** The standard line of LBP calculated from the UV absorption spectra.
